# Genome-Wide Analysis of Loss of Heterozygosity in Breast Infiltrating Ductal Carcinoma Distant Normal Tissue Highlights Arm Specific Enrichment and Expansion across Tumor Stages

**DOI:** 10.1371/journal.pone.0095783

**Published:** 2014-04-18

**Authors:** Xiaoyang Ruan, Hongfang Liu, Lisa Boardman, Jean-Pierre A. Kocher

**Affiliations:** 1 Biomedical Statistics and Informatics, Department of Health Sciences Research, Mayo Clinic College of Medicine, Rochester, Minnesota, United States of America; 2 Department of Gastroenterology and Hepatology, Mayo Clinic, Rochester, Minnesota, United States of America; Ohio State University Medical Center, United States of America

## Abstract

Studies have shown concurrent loss of heterozygosity (LOH) in breast infiltrating ductal carcinoma (IDC) and adjacent or distant normal tissue. However, the overall extent of LOH in normal tissue and their significance to tumorigenesis remain unknown, as existing studies are largely based on selected microsatellite markers. Here we present the first autosome-wide study of LOH in IDC and distant normal tissue using informative loci deduced from SNP array-based and sequencing-based techniques. We show a consistently high LOH concurrence rate in IDC (mean = 24%) and distant normal tissue (m = 54%), suggesting for most patients (31/33) histologically normal tissue contains genomic instability that can be a potential marker of increased IDC risk. Concurrent LOH is more frequent in fragile site related genes like WWOX (9/31), NTRK2 (10/31), and FHIT (7/31) than traditional genetic markers like BRCA1 (0/23), BRCA2 (2/29) and TP53 (1/13). Analysis at arm level shows distant normal tissue has low level but non-random enrichment of LOH (topped by 8p and 16q) significantly correlated with matched IDC (Pearson r = 0.66, p = 3.5E-6) (topped by 8p, 11q, 13q, 16q, 17p, and 17q). The arm-specific LOH enrichment was independently observed in tumor samples from 548 IDC patients when stratified by tumor size based T stages. Fine LOH structure from sequencing data indicates LOH in low order tissues non-randomly overlap (∼67%) with LOH that usually has longer tract length (the length of genomic region affected by LOH) in high order tissues. The consistent observations from multiple datasets suggest progressive LOH in the development of IDC potentially through arm-specific pile up effect with discernible signature in normal tissue. Our finding also suggests that LOH detected in IDC by comparing to paired adjacent or distant normal tissue are more likely underestimated.

## Introduction

Loss of heterozygosity (LOH) has been shown to be an important genetic event in most types of cancer, and used to infer the genomic location of cancer-related genes [1], [2]. As the most common histological type of breast cancer, infiltrating ductal carcinoma (IDC) accounts for more than 70% of breast invasive carcinoma. Many studies have been conducted to characterize LOH in IDC [3]. Further investigations show LOH is not limited to IDC. Studies on pre-invasive breast lesions, especially ductal carcinoma *in situ* (DCIS), have shown LOH similar to those identified in IDC [4], suggesting DCIS as potential precursor or “marker of increased risk” of IDC [4] and LOH as an important biomarker of premalignant lesion.

The majority of LOH studies on IDC [3], DCIS [4], or breast cancer associated epithelium/stroma [5], [6] detect LOH by comparing with paired adjacent or distant normal tissue. Some studies used blood as primary control, and resorted to normal tissue when blood unavailable [7], [8]. This is based on the assumption that histologically normal tissues are also genetically normal. However, this assumption may not hold, as several lines of evidence shown LOH occurred early in morphologically and histologically normal tissues from breast cancer patients [9]–[11]. For example, Cavalli L. *et al* [9] detected LOH at BRCA1 locus in both IDC and adjacent normal tissue by comparing to peripheral blood in informative patients (i.e. heterozygote in blood) through microsatellite markers. Moinfar F. *et al* [11] found LOH in stromal and epithelial cells either adjacent to or at a distance from foci of IDC or DCIS. Reis-Filho JS *et al* [4] reviewed several independent studies that reported LOH in normal tissue from breast cancer patients. It can be implied from the existing studies that detecting LOH in breast malignant or pre-malignant lesion by comparing to the allelic status in adjacent or even distant normal tissue may underestimate the amount of LOH since LOH may already be present in the normal tissue. However, it is hard to estimate to what extent the LOH might be underestimated, as existing studies on this topic are largely based on microsatellite markers [4], which has very limited genome coverage. For the same reason, there is still a lack of high-resolution view of the extent and frequency of LOH in normal breast tissues from IDC patients and how they might be related to tumorigenesis.

Using informative loci (i.e. heterozygote in blood and homozygote in paired tissue sample) deduced from SNP array-based technique, here we examined LOH across autosomal arms for tumor (Tt) and distant normal tissue (Td) from 33 IDC patients by comparing to paired blood samples. To have a continuous view of the changes in LOH after tumor formation, we examined an independent set of 548 IDC patients representing all stages of IDC. We further analyzed high-density genotyping data derived from whole genome sequencing (40×coverage) of three IDC patients, two for which we have blood, Td, and Tt, and one with blood, Tt, and metastatic tumor (Tmet). Due to the high resolution of the informative loci from sequencing results, we also investigated the fine structure (location, length) of concurrent LOH in paired tissue samples. Specifically, we are interested in the overall extent of LOH in Td, how LOH is enriched at autosomal arm scale, and whether it affects other genomic regions besides of the reported genes like BRCA1/2 and TP53 [9], [10], [12], and how it is inherited/developed in matched IDC.

## Results

### IDC Distant Normal Tissues have Signatures of Arm Specific LOH Enrichment Similar to IDC

We examined autosome-wide the amount of concurrent LOH (≥2 HET->HOM events) in Td and Tt for each individual. For all the LOH detected in Td, averagely 54% (Table S1, Col D) has concurrent LOH in Tt. For all the LOH detected in Tt, averagely 24% (Table S1, Col G) has concurrent LOH in Td. The decrease in concurrent LOH is due to the high proportion of independent LOH in Tt. Noteworthy is that the percentage (24%) also reflects the average proportion of LOH that won’t be detected in IDC if using normal tissue as control. To estimate whether the concurrent LOH is a random effect, we performed permutation tests using sequencing derived LOH for two patients (A7-A0CE, BH-A0B3) that have relatively low LOH concurrence rate among the 33 patients. Permutations (shown in the last section of result) indicate that the observed level of LOH concurrence is a non-random effect, suggesting LOH in Td as biomarker potentially reflecting increased risk of IDC. Exceptions were noted for two patients. One (BH-A0BJ) has zero concurrent LOH and another (BH-A0B2) has much higher level of LOH in Td than in Tt, suggesting their IDC and distant normal tissue have distinct genetic background.

**Figure 1:**
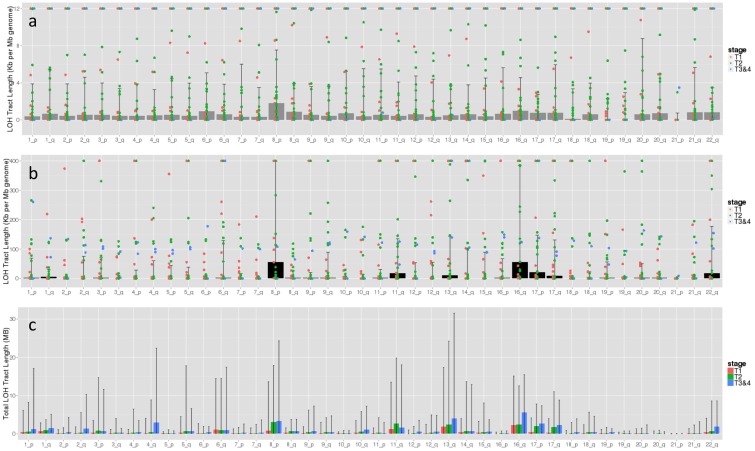
Total LOH tract length (≥2 HET->HOM events) adjusted for chromosome arm length. a) Td, b) Tt of 31 IDC patients. Overlaid scattering points are the actually value for each patient colored by T stage of the patient’s Tt sample. The scale of the Y axis was adjusted to magnify LOH patterns. Scatter points outside of the Y axis top boundary were represented by points at the top of the boundary to show their existence. c) LOH tract length (≥2 HET->HOM events) of 548 IDC patients stratified by T stage. Bar height and error bar represent the median, first and third quartile values across patients.

Analysis of the remaining 31 IDC patients (excluded BH-A0BJ and BH-A0B2) shows arm specific LOH enrichment in Tt, where 8p, 11q, 16q, 17p, and 17q stand out from other arms after adjusting for arm lengths. Tt samples with lower T stage have generally lower levels of LOH (Figure 1b). Td samples of the 31 IDC patients have low level, but non-random LOH enrichment across autosomal arms (peaked at 8p and 16q) (Figure 1a). The LOH enrichment patterns are significantly correlated between Td and Tt (Pearson r = 0.66, p = 3.5E-6). The LOH observed in Td is unlikely caused by contamination for two reasons. First, purity information provided by TCGA shown no presence of tumor nuclei in these Td samples (Table S1). Second, there is no significant difference between Td samples from patients with different stages of Tt (Figure 1a), whereas the Tt samples have significantly more LOH at higher T stages (Figure 1b).

We observed that the majority of the LOH in Td are copy-neutral. Proportionally, they account in average for 99% of all LOH found in each Td sample (SD = 1.5%). It is worth to note that arrayCGH (a method often used to characterize LOH) cannot detect copy-neutral LOH and therefore would not report the presence of this prominent type of LOH in this dataset.

### Genes with Highly Concurrent LOH in IDC and Distant Normal Tissue are Frequently Collocated with Fragile Sites

We examined concurrent LOH (≥2 HET->HOM events) in Td and Tt for each of the 31 IDC patients. Among all autosomal genes, MCPH1, CSMD1, MACROD2, NTRK2 (collocates with FRA9D), WWOX (collocates with FRA16D) topped the list of concurrent LOH, where ≥9 patients have genomic overlapping LOH in Td and Tt samples (Table 1, see Table S5 for full list). Most of the genes with high levels of concurrent LOH are often located within or in the vicinity of known fragile sites. E.g. 49 (11.4%) of the 429 genes that have ≥6 patients with concurrent LOH are collocated with known fragile sites, which is significantly higher than the average level of 8.4% and 8.5% by using all genes and genes that have ≥1 patients with concurrent LOH, respectively (P = 0.02 by goodness of fit test).

**Table 1:** Gene with top concurrent LOH in IDC and distant normal tissue.

Gene	Location	No. patients withconcurrent LOH(w or w/o genomic overlap)^a^	No. patients withconcurrent LOH(with genomic overlap)^b^	No.informative patients^c^	Collocate with fragile site	In cancer gene census
**MCPH1**	*8p23.1*	11	11	31	N	N
**CSMD1**	*8p23.2*	10	10	31	N	N
**MACROD2**	*20p12.1*	10	10	31	N	N
**NTRK2**	*9q22.1*	10	10	30	FRA9D	N
**WWOX**	*16q23.3-q24.1*	11	9	31	FRA16D	N
**DAB1**	*1p32-p31*	10	9	31	FRA1L|FRA1B	N
**RYR2**	*1q43*	10	9	31	N	N
**CDH4**	*20q13.3*	9	9	31	N	N
**CNTN4**	*3p26.3*	9	9	31	N	N
**DLGAP2**	*8p23*	*9*	9	31	N	N
**GAS7**	*17p13.1*	8	7	31	N	Y
**ALK**	*2p23*	7	7	31	N	Y
**FHIT**	*3p14.2*	7	7	31	FRA3B	Y
**PDGFRA**	*4q12*	7	7	31	FRA4B	Y
**NFIB**	*9p24.1*	7	6	31	N	Y
**ABL1**	*9q34.1*	6	6	31	N	Y
**C16orf75**	*16p13.13*	6	6	28	N	Y
**JAZF1**	*7p15.2-p15.1*	6	6	31	N	Y
**LPP**	*3q28*	6	6	31	FRA3C	Y
**RAD51L1**	*14q23-q24.2*	6	6	31	N	Y
**BRCA1**	*17q21*	0	0	23	N	Y
**BRCA2**	*13q12.3*	2	2	29	N	Y
**ATM**	*11q22-q23*	2	2	22	N	Y
**TP53**	*17p13.1*	1	1	13	N	Y
**CDH1**	*16q22.1*	3	3	30	FRA16B|FRA16C	Y
**MAP2K4**	*17p12*	1	1	27	FRA17A	Y

a LOH supported by ≥2 HET->HOM events in both Td and Tt in same gene region but do not require genomic overlap.

b LOH supported by ≥2 HET->HOM events in both Td and Tt and have genomic overlap of at least 1 bp in same gene region.

c Number of patients that have at least 1 heterozygote SNP in the corresponding gene region.

It was previously shown [9], [10], [12] that breast cancer related genes like BRCA1/2 and TP53 have concurrent LOH in breast carcinoma and normal tissue. We also examined the LOH concurrence rate for these genes. Our analysis shows that several well-reported breast cancer related genes including BRCA1/2, CDH1, ATM, TP53, and MAP2K4 have only ≤3 patients with concurrent LOH (Table 1). We further checked the 487 genes documented in cancer gene census [13] for concurrent LOH. The top LOH concurrences were seen for GAS7, ALK, FHIT, and PDGFRA, where ≥6 patients have concurrent LOH (Table 1). However, there is no significant enrichment of cancer related genes among those genes with top LOH concurrence. E.g. 12 (2.8%) out of the 429 genes that have > = 6 patients with concurrent LOH are cancer related genes, which is not significantly higher than the average level of 1.7% and 1.9% by using all genes and genes that have ≥1 patients with concurrent LOH, respectively (P = 0.2 by goodness of fit test).

### Arm-specific LOH Enrichment in an Independent Set of IDC Patients

The arm-specific LOH enrichment was observed in an independent set of 548 IDC patients, which shows LOH enrichment in 8p, 11q, 13q, 16q, 17p, and 17q across all tumor T stages (Figure 1c, Table S2). Noteworthy is that LOH on almost all arms increase with tumor T stage (Figure 1c), indicating growing genome instability as tumor grows. The increase in LOH is observed for all LOH subtypes (i.e. copy neutral/loss/gain) (Data not shown). It is remarkable that these arms contain several breast cancer related genes including ATM, BRCA1/2, CDH1, TP53, MAP2K4 that have LOH been highly reported [3]. LOH of these genes seems to be a result of LOH at the whole arm level, as majority of the genes located on these arms have LOH.

### Deep Sequencing Indicates Non-random LOH Concurrence in IDC and Distant Normal Tissue, and Suggests Possible Extension Effect

To have a detailed view of how LOH in the same genomic region vary between paired IDC and distant normal tissues, we retrieved deep sequencing (40x) results for two IDC patients that have sequenced blood, Td, and Tt. Results for another IDC patient with sequenced blood, Tt, and Tmet were also retrieved. Large variance in LOH was observed for the first two patients, both with grade 3, T2 stage IDC (Table 2). For patient #3, the Tmet sample has higher LOH burden than Tt (Table 2).

**Table 2:** LOH of three patients with deep sequencing results.

Patient	Disease status	Sample	No. HET->HOM events	No. LOH (1 HET->HOM event)	No. LOH (≥2 HET->HOM events)	Total LOH tract length (≥2 HET->HOM events) (KB)
TCGA-A7-A0CE (#1)	SBR Grade 3,T2,Ductal,ER-,PR-,HER2?^a^	Td (L-stage)	5.2 k	4.7 k	240 (237/0/3)^b^ [135]^c^	1.48 k (1.47 k/0/8.3)^b^
		Tt (H-stage)	154.5 k	68.8 k	32.1 k (26.4 k/2.7 k/3.2 k)	57.1 k (47.1 k/2.4k/7.6 k)
TCGA-BH-A0B3 (#2)	SBR Grade 3,T2,Ductal,ER-,PR-,HER2-	Td (L-stage)	12.1 k	9.9 k	954 (949/3/2) [621]	11.4 k (11.2 k/284/11)
		Tt (H-stage)	28.6 k	23.1 k	2.5 k (1.8 k/466/232)	14 k (11.6k/1.7 k/817)
TCGA-E2-A15E (#3)	SBR Grade 3,T1c,Ductal,ER+,PR+,HER2?	Tt (L-stage)	7.0 k	6.3 k	330 (286/9/35) [146]	1.8 k (1.6 k/4.5/143)
		Tmet (H-stage)	49.5 k	31.5 k	7.2 k (3.1 k/3.4 k/606)	12.3 k (5.5 k/3.6 k/3.1 k)

a Question mark represents equivocal or unknown.

b Numbers in parentheses are for copy (neutral/loss/gain) LOH.

c Number of concurrent LOH in H-stage sample.

**Figure 2:**
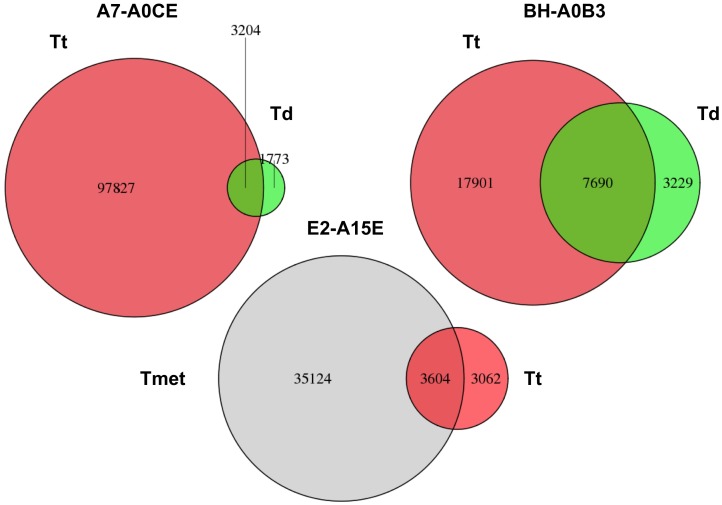
Numbers of concurrent and independent LOH (≥1 HET->HOM event(s)) for three patients with deep sequencing data. See Table 3 for results using stringent criterion (≥2 HET->HOM events).

Analysis of concurrent LOH in L-stage (Td for the first two patients, and Tt for the 3^rd^ patient) and H-stage (Tt for the first two patients, and Tmet for the 3^rd^ patient) samples shows roughly 2/3 of LOH in a L-stage sample have concurrent LOH in corresponding H-stage sample when using both stringent (≥2 HET->HOM events) (Table 2, Col 6) and loose (≥1 HET->HOM events) criteria to define LOH (Figure 2). On the other hand, due to the high percentage of independent LOH, the LOH in H-stage samples have relatively smaller proportion (3%∼30%) of concurrent LOH in corresponding L-stage samples. However, intricately designed permutation (n = 5000) of LOH location indicates the observed LOH concurrence rate as a non-random effect. E.g. permutation of LOH in patient #1 shows 3.1%[Min = 2.2%, Max = 4.1%] and 0.15%[Min = 0.11%, Max = 0.21%] concurrence rate for Td and Tt respectively, which are significantly (p<2.2e-16) lower than the actual observation (64.4%, 3.2%). Similar levels of significant difference between expected and observed concurrence rate was observed for the other two patients using both loss and stringent criterion, which indicates the L-stage samples as potential precursor of corresponding H-stage samples for the current three patients.

Analysis of specific gene regions, e.g. collocated or not collocated with fragile sites, listed or not listed in the cancer gene census, shows non-random LOH concurrence. For simplicity, here we only show the LOH concurrence rate for cancer genes. The three patients respectively have 58, 95, 72 cancer genes with LOH in L-stage samples, and 43 (74%), 71 (75%), 47 (65%) have concurrent LOH in corresponding H-stage samples (Figure S2). A list of the cancer genes with concurrent LOH is tabulated in Table S3. Two cancer genes including FHIT (collocated with FRA3B) and PDGFRA (collocated with FRA4B) that topped the concurrent LOH list in SNP array based analysis (Table 1) also show high levels of concurrent LOH in the sequencing based analysis.

**Figure 3:**
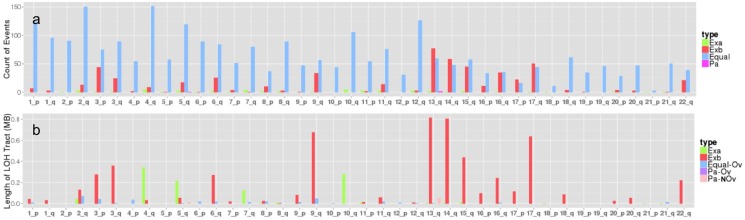
Count and length of concurrent LOH from patient A7-A0CE (#1). a) Count of concurrent LOH. Exa for longer LOH tract length in Td; Exb for longer LOH tract length in Tt; Pa for partial overlap. b) LOH tract length of different categories of LOH. Exa for extension length in Td; Exb for extension length in Tb; Equal for length of equal LOH; Pa-Ov for length of the overlapping part of the partial overlap; Pa-NOv for length of the non-overlapping part of the partial overlap.

The high resolution of informative loci from sequencing data makes it feasible to query the fine structure of concurrent LOH. Supported by the non-random LOH concurrence rate, we assume the L-stage samples as precursor (or marker of increased risk) of corresponding H-stage samples and compared the concurrent LOH in terms of overlap pattern and tract lengths (Figure S1). Among the concurrent LOH, we found that 8%–19% have different tract lengths between H and L-stage samples and most have longer tract lengths in H-stage samples (Table 3). At autosomal arm scale, take patient #1 for example, most arms have stable concurrent LOH (same tract lengths in Td and Tt) (Figure 3a). Among the rest of arms, Td and Tt have their preferred arms for extension (defined in Figure S1), with Tt has longer LOH tract on most arms (Figure 3b, Table S6). Overall, the count of concurrent LOH that have different tract length between L and H-stage samples account for 0.55%–1.4% of all LOH detected in H-stage sample, and explain 10%∼32% of total LOH tract length in IDC and as high as 54% of that in IDC distant normal tissues (Table 3). Analysis across the three patients (Figure 3b, Table S6) indicates that 17q has relatively consistent increases of LOH in the H-stage sample.

**Table 3:** Overlap pattern of concurrent LOH.

Sample	Exa-d	Exa-s	Exa length in MB^a^	Exb-d	Exb-s	Exb length in MB^b^	Partial Overlap	Equal	Total	Prop of change^c^
TCGA-A7-A0CE	0|0^d^	53|2	0.8|0.12 (54%|8%)	93|7	460|23	5.6|0.31 (10%|0.5%)	3|3	2595|100	3204|135	19%|26%
TCGA-BH-A0B3	11|5	254|47	3.2|1.33 (28%|12%)	9|2	351|34	4.5|0.84 (32%|6%)	14|14	7051|519	7690|621	8%|16.4%
TCGA-E2-A15E	2|0	89|3	0.9|0.24 (50%|13%)	81|4	363|20	2.8|0.29 (23%|2%)	11|11	3058|108	3604|146	15%|26%

a Extension length of Exa-d + Exa-s. Number in parentheses is the percentage of total LOH tract length.

b Extension length of Exb-d + Exb-s. Number in parentheses is the percentage of total LOH tract length.

c (Total - Equal)/Total.

d Use LOH supported by ≥1 | ≥2 HET->HOM events.

## Discussion

This is the first study that provided an autosome-wide high-resolution view of the progression LOH in relation to tumorigenesis of IDC, a major histological type of breast cancer. Using informative loci deduced from SNP array-based technique, analysis shows at autosome level non-random LOH concurrence in IDC and distant normal tissue. The observation is in line with previous finding of LOH in IDC adjacent and distant normal tissue [9], [11], indicating histologically normal tissues at a distance from IDC foci are genetically abnormal. On the other hand, the non-random LOH concurrence also suggests that LOH detected using normal tissue as control could be underestimated. Interestingly, we observed a discernible signature of arm specific enrichment of LOH in distant normal tissue that is significantly correlated with the LOH pattern in IDC. The finding revealed that in IDC the preferential enrichment of LOH in 8p, 11q, 13q, 16q, 17p, and 17q, which was mentioned separately in previous studies [3], [4] and independently observed in two datasets in current study, may emerge through a pile up effect that starts early in histologically normal tissue. Noteworthy is that although the pile up of LOH from lower to higher T stage seem to be a result of cell proliferation, its causal relationship with malignant conversion, i.e. from normal to premalignant to malignant cell, is yet to be determined. It is also unclear whether similar pattern of LOH enrichment also exists in breast tissue from women with non-tumor associated breast diseases or healthy women. Nevertheless, the extent of LOH on autosomal arm in normal tissue seems to be an important factor that determined the extent of LOH on the corresponding arm in IDC, suggesting histologically normal tissues contain genomic instability that can be a predictive marker of IDC.

Previous findings show concurrent LOH in BRCA2 and TP53 in breast cancer and adjacent normal tissue [9], [10], [12]. In our dataset, the LOH concurrence rates of the breast cancer related genes are only at low to medium level among genes documented in the cancer gene census [13], and more like a random effect when compared with the levels of concurrent LOH in fragile site genes. Autosome-wide scan shows genes with the top LOH concurrence rate have significant enrichment of fragile site related genes, among which the most interesting signals are WWOX, NTRK2, FHIT, and PDGFRA. They were discussed here because of either be reported as premalignant marker or a non-negligible level of concurrent LOH. WWOX that encompass FRA16D fragile site was shown to play important role in breast cancer tumorigenesis [14]. NTRK2 is collocated with FRA9D, and its value as a premalignant marker deserves future investigation. FHIT and PDGFRA have concurrent LOH been detected in samples that were subject to deep sequencing. FHIT is a gene that encompasses the common fragile site FRA3B on chromosome 3, where genetic alterations including deletion was suggested as a marker of pre-malignant lesion [15]. PDGFRA is collocated with FRA4B and related to tumor progression [16], [17], but less reported as a pre-malignant marker. As we have previously shown several fragile sites actually co-occurs with high homozygosity region in both HapMap healthy samples and NCI-60 cancer cell lines [18], these consistent observations suggest the potentially critical role of fragile site LOH in malignant conversion. Its value in clinical applications like cancer risk assessment deserves further investigation.

Using sequencing-based high resolution LOH, we show that LOH in low order tissues non-randomly overlap with LOH of different tract length, and usually longer, in high order tissues. This indicates that a significant amount of LOH are seeded from genomic regions prone to LOH, despite that the overlap can happen between cells belongs to same clone, or arise from independent LOH in cells with different lineages. The observation coincides with our previous finding that genomic regions with extended length of homozygotes, a product of large region of LOH if not IBD (identical by descent), in cancer cell lines may largely stem from existing high homozygosity regions in healthy state [18]. On the mechanism side, this might be explain by secondary mitotic recombination promoted by increased sequence similarity [19]–[21] in LOH regions, or more frequent LOH-causing genetic disorder (E.g. double strand break, chromosomal deletion) around LOH spots, as LOH is an indicator of genome instability [22], [23].

Our data show discernible global LOH disorders including arm-specific LOH enrichment and fragile site LOH in IDC distant normal tissue, suggesting them as valuable biomarkers of pre-malignant lesion. Noteworthy is that LOH alone may only explain part of cancer predisposition, as other disorders (E.g. germline CNV, epigenetic change) have also been shown to play a role in malignant conversion [24], [25]. Due to the limited annotation information provided by TCGA, the origin (epithelial or stromal) of most solid tissue samples cannot be explicitly determined in this study. Further study utilizing single cell based techniques and multi-focal sampling (e.g. adjacent/distant stromal/epithelial normal) are required to decipher the precedence of these genetic/epigenetic disorders, and also phylogenicity of LOH in malignant conversion and tumor progression, which will provide patients and oncologists valuable diagnostic and prognostic information.

## Materials and Methods

### IDC Patients with Genotyping Results

Analysis was limited to patients with breast infiltrating ductal carcinoma (IDC) histology type. We obtained 33 IDC patients (n = 9, 22, 2 for T1, T2, T3&4, respectively) with paired blood/Td/Tt, as well as an independent set of 548 IDC patients (n = 154, 332, 62 for T1, T2, T3&4, respectively) with paired blood/Tt from TCGA project (http://cancergenome.nih.gov/). The patients were selected based on available purity information from TCGA so that the Td samples have zero percentage of tumor nuclei and Tt samples have > = 60 percentage of tumor nuclei (Table S1). Of the 32 patients with information on the distance from Td to Tt, all have distal (>2 cm) normal. Histological figures of the microdissected tissue samples are publicly available at http://www.cbioportal.org/public-portal/. All samples were genotyped on Affymetrix SNP 6.0 array. Genotypes were called by using BirdSuite (version 1.5.5), which estimates allele specific genotype by considering both common and rare copy number variation (CNV) regions [26]. Affymetrix annotation file (genome version NCBI36/hg18) was used to annotate the probe-set. 273 SNPs with call rate <95% were excluded from analysis. All samples have SNP call rate > = 95%. The average no-call rate was 0.44% for blood samples, and 1.65% for tumor samples (1.34% in T1, 1.78% in T2, and 1.74% in T3&4).

### IDC Patients with Sequencing Results

Whole genome sequencing bam files of 3 IDC patients with paired germline/Td/Tt (or germline/Tt/Tmet) were obtained from TCGA cancer genomics hub (https://cghub.ucsc.edu/). The average coverage is 40× ranging from 25× to 56×. Raw bam files were realigned on the reference genome (version GRCh37/hg19) by using GATK (version 1.7) [27]. SNP was called by running the GATK multiple sample genotype call and quality calibration procedure. This resulted in 5,236,586 biallelic SNPs with a “pass” tag in the quality field, and has reference SNP number (dbSNP137). Insertion/deletion polymorphism (INDEL) was called by using DINDEL (version 1.01) [28] using the realigned bam files as input. Out of 1,194,452 calls, 792,607 biallelic INDELs that passed quality check and documented in dbSNP137 were also used. SNP and INDEL variants with no-call ≥3 across the 9 samples were removed, which left 5,900,038 effective variants and an average 0.46% no-call rate. Copy number variation (CNV) was detected by cnvnator (v0.2.7) [29] using bin size of 1000. We removed CNV calls less than 100 k in length to improve the true positive rate. A summary of the sample information is provided in Table S4.

### Gene Location

Genomic location of genes in bed format was obtained from the UCSC website (http://genome.ucsc.edu/cgi-bin/hgTables). Versions NCBI36/hg18 and GRCh37/hg19 were used for datasets A and B, respectively, based on the version of annotation file/reference genome being used. The UCSC gene identification was then mapped to the HGNC gene name. For genes with multiple entries, the longest one was used.

### Cancer Gene List

The cancer gene list was obtained from the cancer gene census (version 2012-3-15) [13], which includes 487 cancer genes for which mutations have been causally implicated in cancer.

### Fragile Site Database

Fragile site database was downloaded from HGNC http://www.genenames.org/cgi-bin/hgnc_stats. A total of 117 fragile sites were use for analysis.

### LOH, LOH Tract Length, and Concurrent LOH

**Figure 4:**
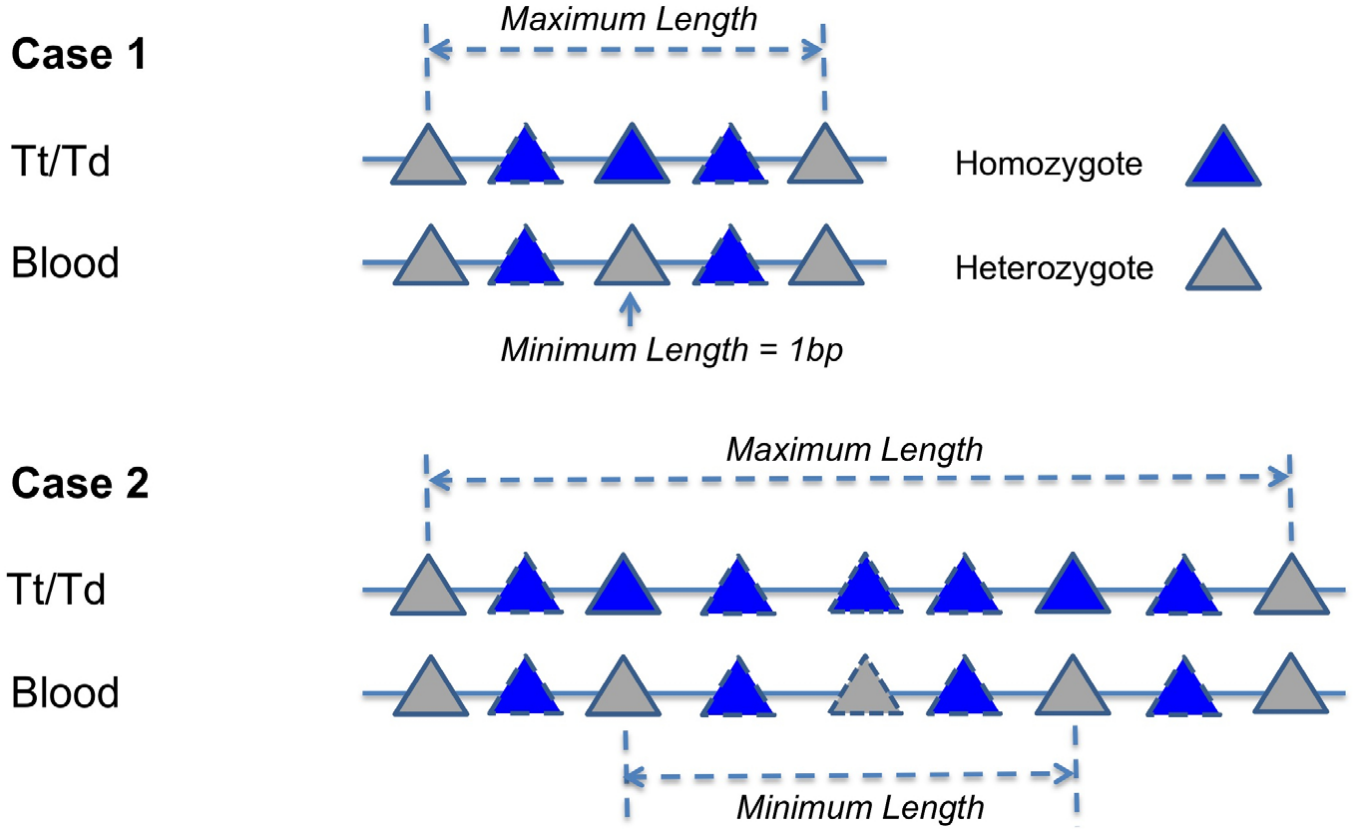
Define LOH and LOH tract length. Case 1) LOH composed of 1 HET->HOM event. The minimum LOH tract length is 1 bp. Case 2) LOH composed of ≥2 HET->HOM events. The minimum LOH tract length is the distance between leftmost and rightmost informative SNPs.

LOH analysis in this study was limited to autosomal chromosomes. Depending on the copy number state, there are copy-neutral, copy-loss, and copy-gain LOH. In conjunction with copy number information, LOH and its subtypes can be detected by tracking genotype changes in SNP(s) that evolve from being heterozygote(s) (HET), also called informative marker(s), in germline DNA (i.e. blood) to homozygote(s) (HOM) in tissue (either tumor or distant normal tissue). We thus use the annotation HET->HOM to describe LOH. Correspondingly, the length of genomic region affected by HET->HOM event(s) is recognized as LOH tract length (Figure 4). LOH can be composed of only one HET->HOM event (LOH tract length = 1 bp), or a stretch of HET->HOM events that can be separated by HOM->HOM without being interrupted by the following transitions: i) HET->HET, ii) HET->UNK (UNK refers to no-call genotype), iii) UNK->UNK, iv) UNK->HET, and v) biallelic mutation (eg. AA->BB). For the latter case, the LOH tract length is computed as the number of base pairs separating the left- and rightmost HET->HOM events (Figure 4). As LOH composed of only one HET->HOM event has potentially high false positive rate, we performed all analyses by primarily using stringent (use LOH supported by ≥2 HET->HOM events) criteria, and attached the result using loose (use LOH supported by ≥1 HET->HOM events) standard where necessary. LOH are detected separately according to CNV status. A typical example of copy neutral LOH is illustrated in Figure S3. LOH with >20% monoallelic mutation (AA->AB) or >20% no-call rate in either germline or tissue are considered low quality and excluded. These account for 1% and 1.3% of all LOH tracts for datasets A and B, respectively.

When analyzing results from genotyping platforms, we accounted for SNP sparsity by splitting copy-neutral LOH >40 Kbp apart and calling the region in between as not affected by LOH. This threshold was derived from the distribution of base pair distance between two heterozygous SNPs in 171 HapMap CEU samples genotyped on the same platform. The 40 kb threshold is close to the upper outer fence (3^rd^ Quantile + 3*IQR) of the distribution.

Specifically, For the 33 IDC patients with germline/Tt/Td we computed LOH by pairing germline to Td, and pairing germline to Tt. For the 548 IDC patients we computed LOH by pairing germline to Tt. Similarly, for the 3 patients with sequencing results, LOH of the first two patients were computed by pairing germline to Td (G-Td), and germline to Tt (G-Tt). For the third patient, LOH were computed by pairing germline to Tt (G-Tt) and germline to Tmet (G-Tmet). Concurrent LOH is called when LOH in paired tissue samples for one patient have genomic overlap of at least 1 bp.

### Overlapping Pattern of Concurrent LOH in Paired Samples

For the three IDC patients with sequencing data, we examined overlap patterns of concurrent LOH in Td and Tt (the first two patients) as well as in Tt and Tmet (the third patient). We considered three major classes of overlap (Figure S1):

1. Equal (Eq) when concurrent LOH have the same start and end position.
2. Extension (Ex) that has two sub classes
  - 2a) Extension is observed in the lower stage sample (Exa) when concurrent LOH have same start or end position but the lower stage sample has longer LOH tract length.
  - 2b) Extension is observed in the higher stage sample (Exb) when concurrent LOH have same start or end position but the higher stage sample has longer LOH tract length.
3. Partial overlap (Pa) when concurrent LOH have different start and end position, but overlap at least 1 bp.

To assess if the LOH concurrence is purely random, we performed permutation test. LOH location was shuffled along the autosomal arms except within 3 Mb of the centromere and the 10 Kb region at the end of each arm. During the randomization, if two LOH overlapped, they were merged into a single one. This reduced the number of LOH by 1% to ∼3%, which has no direct impact on the results. 5000 permutations were performed for G-Td and G-Tt of the first two patients, as well as G-Tt and G-Tmet of the third patient, respectively. The distribution of the LOH concurrence rate derived from the permutation test was used as background distribution for the significance test.

### Ethics Statement

Collection of genotype and clinical data from subjects was performed in accordance with TCGA guidelines and regulations. Approval for the study was obtained from the Mayo Clinic Institutional Review Board.

## Supporting Information

Figure S1 **Inheritance of LOH assuming Td as precursor (or marker of increased risk) of Tt.** a) Appearance and hypothetical extension of LOH. b) Possible patterns of concurrent LOH. Green and red lines represent LOH in low- and high-stage samples. Exa and Exb indicate extension in low- and high-stage sample; -s and -d represent single- and double-end extension, respectively. The double-headed arrow in combination 3 and 5 exemplifies the extension length of Exa and Exb, respectively. Pa-Ov represents length of the overlapping part of the partial overlap; Pa-NOv represents length of the non-overlapping part of the partial overlap.
(TIFF)

Figure S2 **Number of cancer genes with overlapping and independent LOH tract.**
(TIFF)

Figure S3 **A typical example of copy neutral LOH in Td and Tt.** Figures from left to right panel show Log R Ratio and B Allele Frequency of blood, Td, and Tt. Heterozygotes were missing in ∼250 kb region in both Td and Tt with no change in Log R Ratio.
(TIFF)

Table S1 **Clinical information and LOH concurrence rate of 33 IDC patients.**
(XLSX)

Table S2 **Arm scale LOH tract length of tumor samples from 548 IDC patients.**
(XLSX)

Table S3 **Gene region concurrent LOH of 3 IDC patients with sequencing results.**
(XLSX)

Table S4 **Summary of whole genome sequencing data of 3 IDC patients.**
(XLSX)

Table S5 **Comprehensive list of genes and associated LOH concurrence rate in 31 IDC patients.**
(XLSX)

Table S6 **Length of concurrent LOH of 3 IDC patients with sequencing results.**
(XLSX)
