# Supplementary figures and images for: Genome-Wide Analysis of Loss of Heterozygosity in Breast Infiltrating Ductal Carcinoma Distant Normal Tissue Highlights Arm Specific Enrichment and Expansion across Tumor Stages
Source: PLoS One. 2014 Apr 18;9(4):e95783. doi: 10.1371/journal.pone.0095783 (PMC3991715; doi:10.1371/journal.pone.0095783)

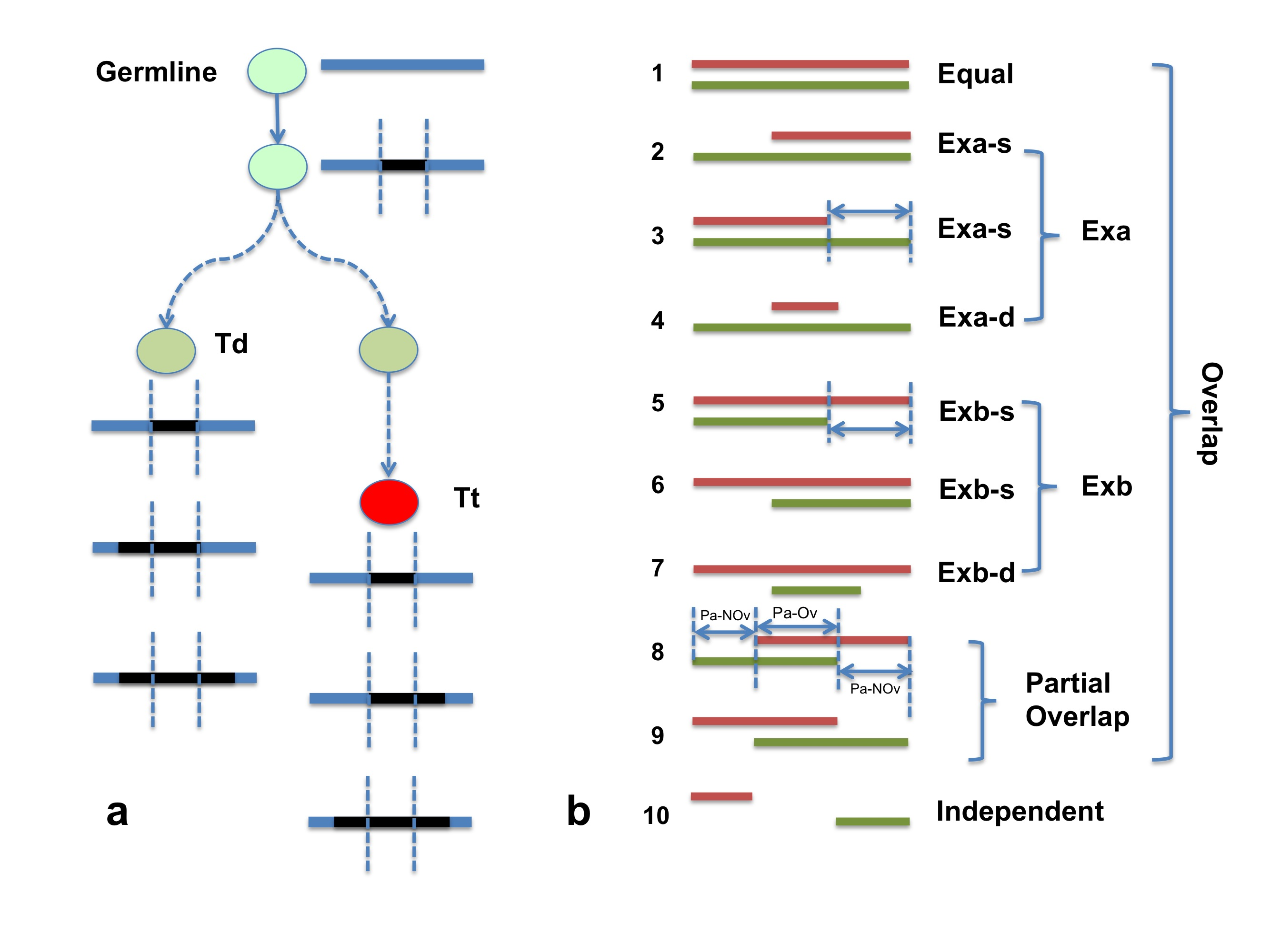

Supplement: Figure S1 — Inheritance of LOH assuming Td as precursor (or marker of increased risk) of Tt. a) Appearance and hypothetical extension of LOH. b) Possible patterns of concurrent LOH. Green and red lines represent LOH in low- and high-stage samples. Exa and Exb indicate extension in low- and high-stage sample; -s and -d represent single- and double-end extension, respectively. The double-headed arrow in combination 3 and 5 exemplifies the extension length of Exa and Exb, respectively. Pa-Ov represents length of the overlapping part of the partial overlap; Pa-NOv represents length of the non-overlapping part of the partial overlap. (TIFF) [file pone.0095783.s001.tiff]

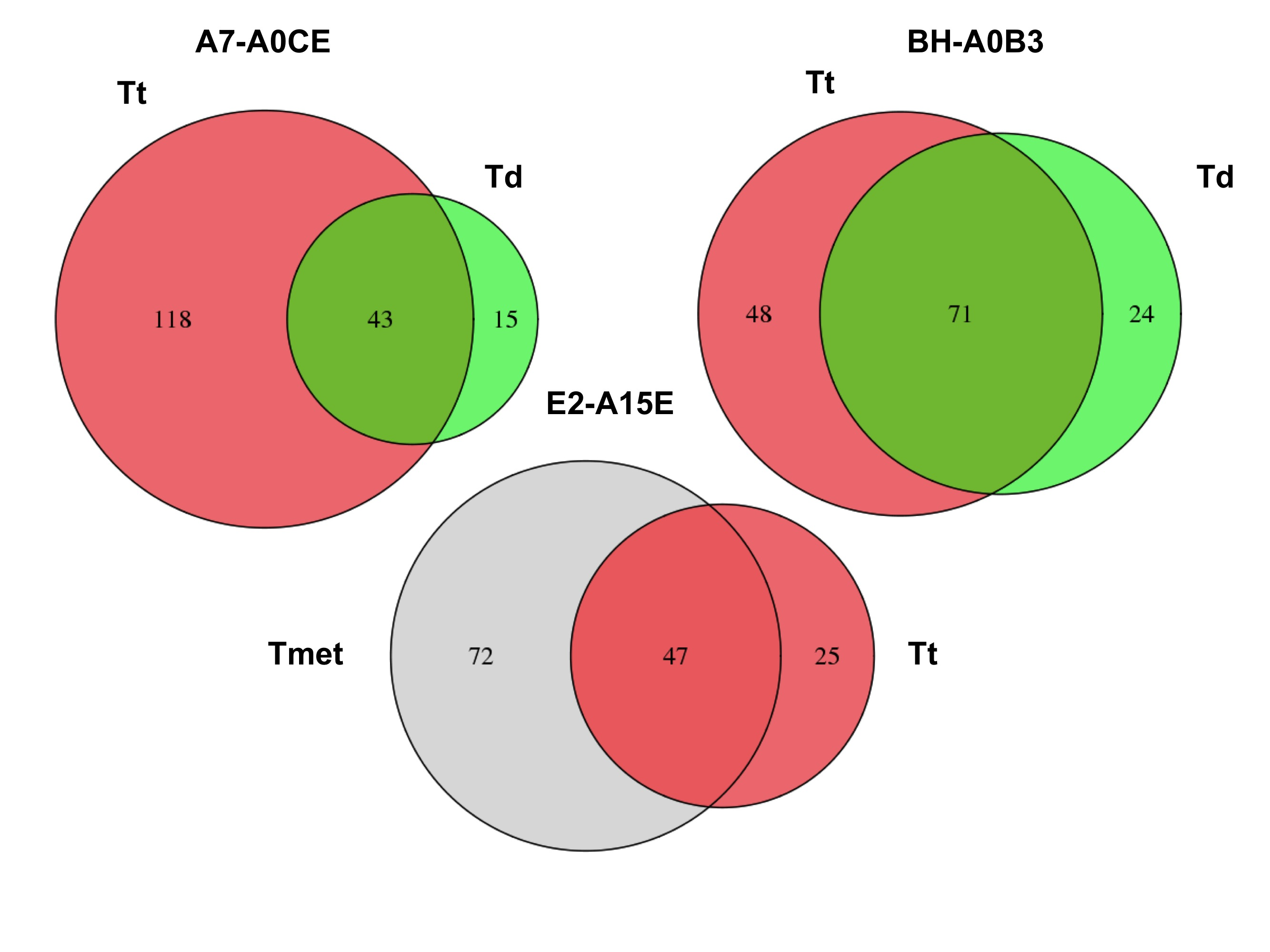

Supplement: Figure S2 — Number of cancer genes with overlapping and independent LOH tract. (TIFF) [file pone.0095783.s002.tiff]

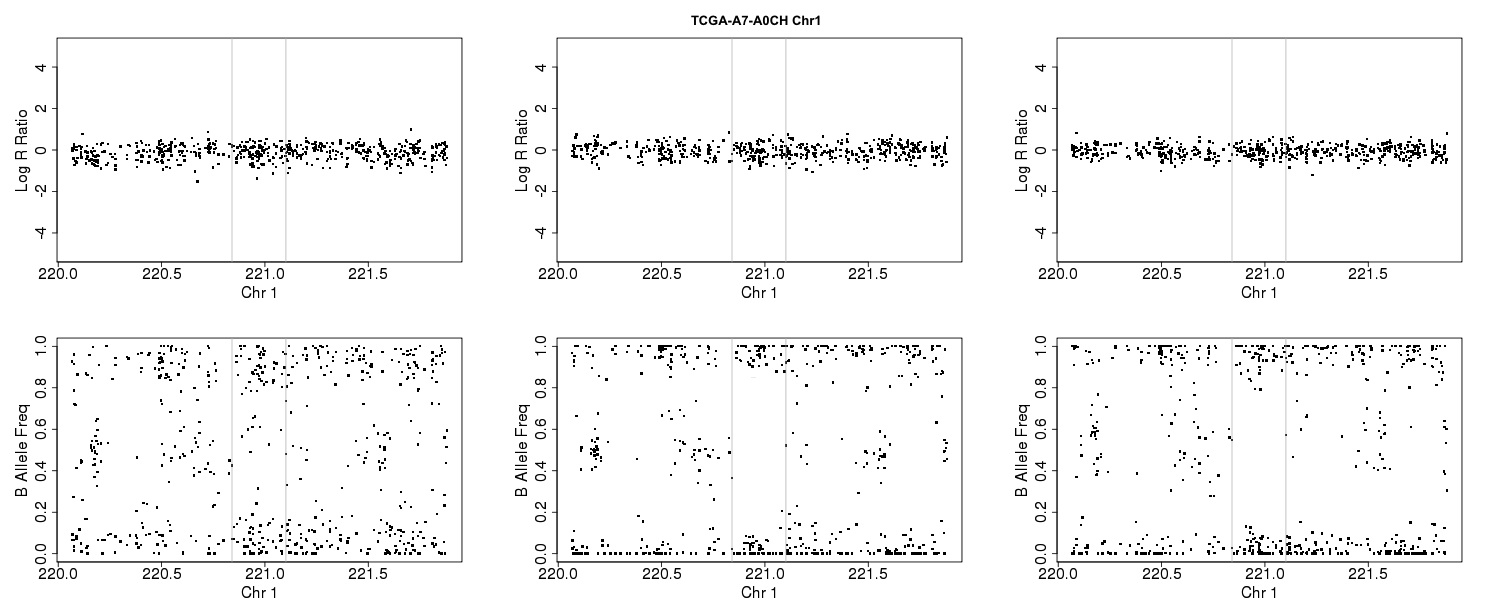

Supplement: Figure S3 — A typical example of copy neutral LOH in Td and Tt. Figures from left to right panel show Log R Ratio and B Allele Frequency of blood, Td, and Tt. Heterozygotes were missing in ∼250 kb region in both Td and Tt with no change in Log R Ratio. (TIFF) [file pone.0095783.s003.tiff]
